# Ferroptosis-Related Genes in Lung Adenocarcinoma: Prognostic Signature and Immune, Drug Resistance, Mutation Analysis

**DOI:** 10.3389/fgene.2021.672904

**Published:** 2021-08-09

**Authors:** Ziyuan Ren, Minghui Hu, Zhonglin Wang, Junpeng Ge, Xiaoyan Zhou, Guoming Zhang, Hongying Zheng

**Affiliations:** ^1^Basic Medical School, Cheeloo College of Medicine, Shandong University, Jinan, China; ^2^Clinical Lab, The Affiliated Hospital of Qingdao University, Qingdao, China; ^3^Department of Mathematics, University of California, Irvine, Irvine, CA, United States; ^4^Department of Biology Engineering, Shandong Jianzhu University, Jinan, China

**Keywords:** ferroptosis, lung adenocarcinoma, prognosis, tumor immune microenvironment, drug resistance, somatic mutation, copy number variation, somatic mutation

## Abstract

It is reported that ferroptosis has close relation with tumorigenesis and drug resistance. However, the clinical significance of ferroptosis in lung adenocarcinoma (LUAD) remains elusive, and the potential targets for ferroptosis-based treatment are limited. In this study, we constructed a 15-gene prognostic signature predicting overall survival based on ferroptosis-related genes (ferroptosis driver genes *VDAC2*, *GLS2*, *FLT3*, *TLR4*, *PHKG2*, phosphogluconate dehydrogenase (*PGD*), *PANX1*, *KRAS*, *PEBP1*, *ALOX15*, and *ALOX12B*, and suppressor genes *ACSL3*, *CISD1*, *FANCD2*, and *SLC3A2*) in The Cancer Genome Atlas (TCGA)-LUAD cohort. The signature’s predictive ability was validated in the GSE68465 and GSE72094 cohorts by survival analysis and independent prognostic analysis with clinical features. Nomograms were provided for clinical reference. Functional analysis revealed that ferroptosis was closely related to cell cycle, cell metabolism, and immune pathways. Pan-cancer analysis comprehensively analyzed these 15 genes in 33 cancer types, indicating that the heterogeneity of 15 genes was evident across different cancer types. Besides, these genes were critical regulators modulating drug resistance, tumor microenvironment infiltration, and cancer stemness. Then, we screened 10 genes (*TLR4*, *PHKG2*, *PEBP1*, *GLS2*, *FLT3*, *ALOX15*, *ACSL3*, *CISD1*, *FANCD2*, and *SLC3A2*) as potential targets for further research because their biological functions in ferroptosis were consistent with their prognostic significance. Somatic mutation and copy number variation analysis revealed that the alteration rates of *KRAS*, *PGD*, and *ALOX15* were more than 1% and significantly associated with overall survival in LUAD. Moreover, the expression of *KRAS* and *PGD* was positively related to tumor mutation burden, indicating that *KRAS* and *PGD* could serve as novel biomarkers for predicting immunotherapy response rate. Our study identified and validated a ferroptosis-related gene signature for LUAD, provided a 10-gene set for future research, and screened *KRAS* and *PGD* as potential novel immunotherapy biomarkers.

## Graphical Abstract

Ferroptosis genes in lung adenocarcinoma.

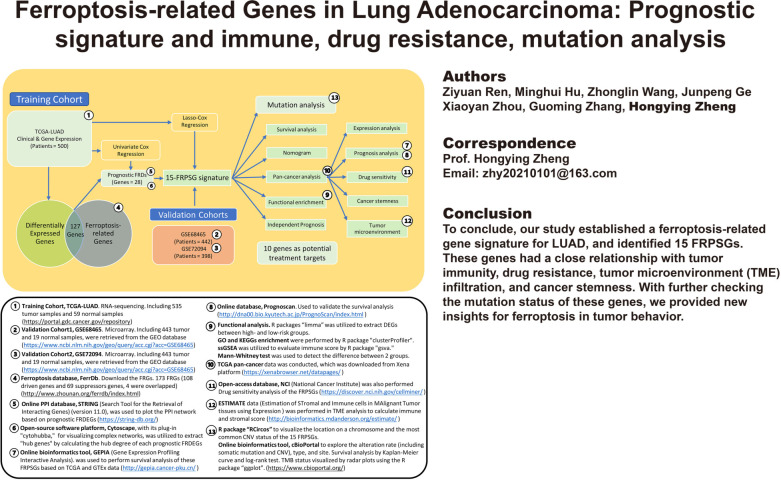

## Introduction

Lung cancer would cause 2.1 million new cases and 1.8 million deaths in 2018, as estimated (Bray et al., 2018). Lung adenocarcinoma (LUAD), one of the non-small cell lung carcinomas (NSCLCs), comprises ∼50% of lung cancer reported cases and shows a continuous upward trend (Imielinski et al., 2012; Kinoshita et al., 2016). Because of the occult nature of early LUAD, most patients are diagnosed with advanced stage or distant metastasis has occurred, losing the opportunity for surgery (Kocher et al., 2015; Siegel et al., 2018). Although some novel regimens such as immune therapy were developed, the 5-year survival rate varies from 4 to 17% (Hirsch et al., 2017). Therefore, exploring novel prognostic signatures and treatment targets will be beneficial.

Ferroptosis is a newly discovered programmed cell death form, promoting reactive oxygen species (ROS) accumulation by glutathione (GSH) consumption and glutathione peroxidase 4 (GPX4) inactivation, which results in subsequent cell death (Xie et al., 2016; Stockwell et al., 2017). Latest studies have demonstrated that ferroptosis impacts cell metabolism, redox state, degenerative diseases, and ischemic reperfusion injury (Stockwell et al., 2017). As for cancers, first, ferroptosis has close relation with the malignant progression of various cancer types (Torti et al., 2018; Mou et al., 2019). The genes that regulate iron metabolisms, such as *NFS1* (NFS1 cysteine desulfurase) (Alvarez et al., 2017) and *CISD1* (CDGSH iron–sulfur domain 1) (Yuan et al., 2016), may become novel targets for tumor-targeted therapy. Second, ferroptosis can reverse the patients’ chemoradiotherapy resistance. Roh et al. found that cisplatin can promote head and neck cancer cell ferroptosis without significant necrosis and apoptosis changes (Roh et al., 2016). Lei et al. (2020) reported that cancer cells were more sensitive to radiotherapy after the inhibition of SLC7A11 and GPX4. Third, tumor immunotherapy has been extensively developed, especially the appearance of immune checkpoint inhibitors. Wang et al. (2019b) discovered that after implementing the immune checkpoint inhibitor, the ferroptosis-specific lipid peroxidation was significantly increased. After blocking the ferroptosis process, the sensitivity to immunotherapy was significantly reduced, indicating that the combination of immunotherapy and inducing ferroptosis treatment is promising (Wang et al., 2019b).

The inhibition of ferroptosis in lung cancer has gained meaningful evidence. Compared with other tissues, lung tissue is in an environment with high oxygen concentration (Valavanidis et al., 2013). This unique environment makes lung tissues need to withstand much oxidative pressure (Sunnetcioglu et al., 2016; Zalewska-Ziob et al., 2019). Therefore, to avoid the facilitated and enhanced ferroptosis during the transformation process, lung cancer cells take various measures to increase the induction threshold of ferroptosis, which leads to the low occurrence of ferroptosis, including upregulation of GSH synthesis (Ji et al., 2018), reductions in iron (Kukulj et al., 2010), inhibition of lipid synthesis (Jiang et al., 2017), compensating the missing enzymatic system GPX4 (Bersuker et al., 2019), and some others (Wang et al., 2019a). Novel regimens for lung cancer based on promoting ferroptosis have also been proposed. Villalpando-Rodriguez confirmed combined use of siramesine and lapatinib could induce ferroptosis in lung cancer cells by inhibiting heme oxygenase-1 (*HO-1*) expression (Villalpando-Rodriguez et al., 2019). However, the clinical significance of ferroptosis in LUAD remains elusive, and the potential targets for ferroptosis treatment are limited.

We established a novel ferroptosis-related 15-gene prognostic signature in The Cancer Genome Atlas (TCGA)-LUAD cohort and validated it in the GSE68465 and GSE72094. Survival analysis and independent prognostic analysis acted mutually to examine the reliability of our signature. Then, we constructed our prognostic signature nomogram to provide a clinical reference for clinicians. Furthermore, functional analysis revealed the relationship between ferroptosis and cell cycle, cell metabolism, and immune response. The pan-cancer analysis provided a comprehensive and systematic characterization of these 15 genes in all 33 cancer types. The results showed that these genes had a close relationship with tumor immunity, drug resistance, tumor microenvironment (TME) infiltration, and cancer stemness, providing new insights for ferroptosis in tumor behavior. Among these 15 genes, 10 genes could be potential treatment targets for basic researches in LUAD because their biological functions in the process of ferroptosis were consistent with their prognostic significance. We performed mutation analysis to explore the alteration and function of these 15 genes in tumor development at the level of genomics. To conclude, our study identified and validated a ferroptosis-related gene (FRG) signature for LUAD, provided a 10-gene set for future research, and screened *KRAS* and phosphogluconate dehydrogenase (*PGD*) as potential novel immunotherapy biomarkers.

## Materials and Methods

### Data Collection

Preprocessed RNA-sequencing (RNA-seq) by fragments per kilobase of exon model per million mapped fragments (FPKM) normalization was downloaded from the TCGA database^1^ LUAD project, including 535 tumor samples and 59 normal samples. The corresponding clinical data were also retrieved from the LUAD project, including 500 patients [13 excluded due to lack of overall survival (OS) and 2 excluded due to lack of RNA-seq] as train cohort. The normalized series matrixes of two total-RNA microarrays, GSE68465 (443 tumor samples and 19 normal samples)^2^ and GSE72094 (442 tumor samples),^3^ were retrieved from the Gene Expression Omnibus (GEO) database (Shedden et al., 2008; Schabath et al., 2016). The corresponding clinical data were also retrieved (GSE68465: 442 patients; GSE72094: 398 patients). The RNA-seq of TCGA and series matrixes of GSE68465 and GSE72094 were processed by “log2(normalized gene expression + 1).” All data analyzed in this study were publicly accessible. No ethics committee approval was required. The policies and publication guidelines of the TCGA and GEO databases were strictly followed.

Then, we searched the ferroptosis database, FerrDb,^4^ to download the FRGs (Zhou and Bao, 2020). A total of 173 FRGs (108 driven genes and 69 suppressors genes, 4 were overlapped) were retrieved and provided in Supplementary Table 1.

### Construction and Validation of the Model

We utilized the function “Wilcox.test” of R package “limma” to extract the ferroptosis-related differentially expressed genes (FRDEGs) between tumor and normal tissues by Mann–Whitney test. Then, we executed the univariate Cox regression to detect the prognostic significance of all FRGs based on OS in the TCGA cohort. Then, the FRDEGs and prognostic FRGs were intersected. The overlapped genes were named prognostic FRDEGs. An online protein–protein interaction (PPI) database, STRING (Search Tool for the Retrieval of Interacting Genes) (version 11.0), was used to plot the PPI network based on prognostic FRDEGs (Szklarczyk et al., 2019). The settings defaulted (network type: full network; active interaction sources: Textmining, Experiments, Database, Co-expression, Neighborhood, Gene Fusion, and Co-occurrence; minimum required interaction score: 0.4). An open-source software platform for visualizing complex networks, Cytoscape with “cytohubba,” was utilized to extract “hub genes” by calculating the hub degree of each prognostic FRDEGs (Shannon et al., 2003). “Cytohubba” is a novel Cytoscape plugin, measuring nodes by their positions in the network to predict their significance. Besides, “cytohubba” identified the crucial elements of biological networks. The topological analysis method was “Degree” (Degree(node) = | the collections of its neighbors|) (Chin et al., 2014). We extracted the nodes with a degree higher than three as “hub genes.” We calculated the Pearson correlation between each two of the prognostic FRDEGs to explore the intrinsic relationship among these genes (the cutoff Pearson correlation coefficient is 0.2). Among these prognostic FRDEGs, only protein-coding genes remained for further study. The R package “glmnet” was utilized to extract prognostic FRDEGs for further multivariate Cox regression by the LASSO (least absolute shrinkage and selection operator) algorithm to minimize overfitting. The selected prognostic FRDEGs were considered as ferroptosis-related prognostic signature genes (FRPSGs). A multivariate prognostic model was constructed by multivariate Cox regression. An online bioinformatics tool, GEPIA (Gene Expression Profiling Interactive Analysis), was used to perform survival analysis of these FRPSGs based on TCGA and GTEx data (Tang et al., 2017). Another online database, Prognoscan, was used to validate the survival analysis (Mizuno et al., 2009). The risk score was calculated with the formula: Risk score = sum {gene expression [log2(normalized gene expression + 1)] × coefficient}. Then, this model was applied in the GSE68465 and GSE72094 cohorts. We stratified patients into high- and low-risk groups by median risk score, respectively. Survival analysis between the two groups was performed by R package “surviminer” using Kaplan–Meier curve and log-rank test. Receiver operator characteristics (ROC) curve was plotted by R package “timeROC” to test the model’s predictive ability. Principal component analysis (PCA) and t-distributed stochastic neighbor embedding (t-SNE) were performed to detect internal characteristics in these two groups by R packages “stats” and “Rtsne.” Independent analysis for risk score was performed by uni- and multivariate Cox regression. Gender, age, stage, T (tumor volume stage), N (lymph node stage), smoking history, and risk score were included in the TCGA cohort. M (metastasis stage) was excluded due to so many missing values (NA) > 30%. Gender, age, T, N, and smoking history were included in GSE68465. Gender, age, smoking history, stage, *KRAS*, *EGFR*, and *TP53* mutation were included in GSE72094.

### Construction of the Nomogram

We also constructed our prognostic gene signature nomogram using the R language. All the prognostic factors included in the multivariate Cox regression of the independent prognosis analysis were included in the nomograms. The calibration curve was plotted to evaluate the fitting and predictive ability of our prognostic model.

### Functional Analysis

We hypothesized that the ferroptosis level was different between the high- and low-risk groups. We compared the 15 FRPSG expressions between the two groups to prove this point. To explore the potential biological processes influenced by ferroptosis between the high- and low-risk groups, we utilized the R packages “limma” to extract DEGs between high- and low-risk groups in the three cohorts for functional analysis. For TCGA cohort, function “Wilcox.test” was utilized (Mann–Whitney test) and | log2FC| ≥ 1 and adj.*P* (adjusted *P*-value) < 0.05 were set as cutoff value. In the GSE68465 cohort, functions “lmFit” and “eBayes” were utilized, and | log2FC| ≥ 0.5 and adj.*P* < 0.05 were set as cutoff value. In the GSE72094 cohort, functions “lmFit” and “eBayes” were utilized, and | log2FC| ≥ 0.9 and adj.*P* < 0.05 were set as cutoff value. Gene Ontology (GO; Ashburner et al., 2000) and Kyoto Encyclopedia of Genes and Genomes (KEGGs; Kanehisa and Goto, 2000) enrichment were performed by R package “clusterProfiler.” Single-sample gene set enrichment analysis (ssGSEA) was utilized to evaluate each group’s immune score by R package “gsva.” Mann–Whitney test was used to detect the difference between the three groups. The detailed annotation table of ssGSEA is presented in Supplementary Table 2.

### Pan-Cancer Analysis of the FRPSGs

To comprehensively analyze the expression and function of FRPSGs in all cancer types, pan-cancer analysis based on TCGA pan-cancer data was conducted, which was downloaded from the Xena platform,^5^ including RNA-seq, clinical data, and stemness scores (Goldman et al., 2020). A total of 33 cancer types were included (the full names and abbreviations are provided in Supplementary Table 3). Expression analysis (Mann–Whitney test) and survival analysis (univariate Cox regression) of the FRPSGs were performed based on TCGA pan-cancer data. We utilized the National Cancer Institute (NCI)-60^6^ database to perform the drug sensitivity analysis of the FRPSGs. NCI-60 is an open-access database based on nine cancer types and 60 cancer cell lines, consisting of mRNA expression level and corresponding z scores of cell sensitivity data (GI50) after drug treatment. We calculated the Pearson correlation between each FRPSG expression and the GI50 to explore the association between FRPSGs and drug sensitivity. We selected 262 Food and Drug Administration-approved drugs or drugs that are currently in clinical trials in this drug sensitivity analysis (Zhang et al., 2020). TME analysis was performed by the Estimation of STromal and Immune cells in MAlignant Tumor tissues using Expression data (ESTIMATE) immune and stromal score downloaded from ESTIMATE^7^ (Yoshihara et al., 2013). Furthermore, to validate the FRPSGs’ immune function, the six immune subtypes were utilized to test the relation between FRPSGs and immune subtypes by analysis of variance (Thorsson et al., 2018). Malta et al. (2018) established a novel index to evaluate the cancer stemness based on one-class logistic regression machine learning algorithm (OCLR). The OCLR extracts transcriptomic (mRNA expression) and epigenetic (DNA methylation pattern) features originated from non-transformed pluripotent stem cells and their differentiated progeny. This cancer stemness feature has been widely used in the computational biological analysis (Liu et al., 2021; Wang et al., 2021). Given that ferroptosis is a crucial regulator of tumor heterogeneity (Li et al., 2021), we calculated the Spearman correlation between the cancer stemness indexes (RNA stemness score, RNAss; DNA stemness score, DNAss) and each FRPSG to explore whether FRGs influence cancer stemness (*P* < 0.05). Furthermore, we utilized the online bioinformatics tool, GEPIA, to explore the correlation between the ferroptosis suppressor genes in FRPSGs and two previously discovered cancer stemness cell biomarkers [CD133 (Bertolini et al., 2009) and CD44 (Leung et al., 2010)] to validate the cancer stemness analysis. The gene expression was normalized by a housekeeper gene (GAPDH) (Schmittgen and Livak, 2008).

### Somatic Mutation and Copy Number Variation Analysis

To fundamentally understand the ferroptosis process during tumor development, we performed somatic mutation and copy number variation (CNV) analysis to detect the alterations of the 15 FRPSGs in LUAD. We utilized the R package ‘‘RCircos’’ to visualize the location on a chromosome and the most common CNV status of the 15 FRPSGs. We utilized an online bioinformatics tool, cBioPortal,^8^ to explore the alteration rate (including somatic mutation and CNV), type, and site in LUAD datasets. Genes with an alteration rate of more than 1% remained for further research. Survival analysis between the unaltered group and altered group was performed using cBioPortal by Kaplan–Meier curve and log-rank test. Genes with a statistically significant prognostic value remained for further research. We explored the association between gene expression and alteration status using cBioPortal. Tumor mutation burden (TMB) has been discovered by a previous retrospective study that showed a high correlation with response to immune checkpoint blockade therapy (Marabelle et al., 2020). We calculated the Spearman correlation between TMB status and gene expression in pan-cancer data to explore novel biomarkers for predicting immune therapy response rate. TMB status was retrieved from cBioPortal (February 6, 2021). A radar plot was presented to visualize the results using the R package “ggplot.”

### Statistical Analysis

The descriptive analysis of each cohort was performed by Chi-square test, Somer’s d test, and independent-samples *t*-test in SPSS 26.0 (International Business Machines Corporation, Armonk, NY, United States). All the statistics, except descriptive analysis, were conducted by R language (version 4.0.3) (R Core Team, 2013). All the adj.*P* [or false rate discovery (FDR)] were adjusted by Benjamini–Hochberg (BH). Adj.*P* < 0.05 was considered as statistically significant.

## Results

### Identification of the Prognostic FRDEGs

**FIGURE 1:**
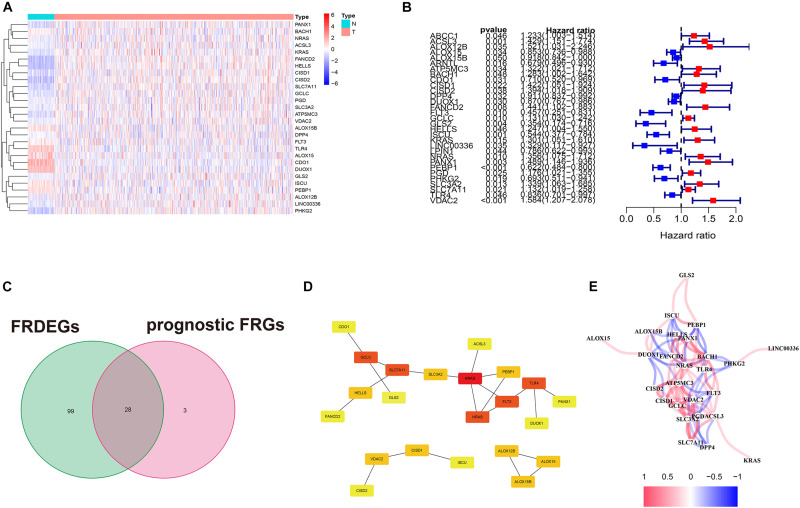
Extraction of prognostic FRDEGs. **(A)** The heatmap of FRDEGs between normal and tumor samples. **(B)** The forest plot of univariate Cox regression. **(C)** The intersection of FRDEGs and prognostic FRGs by univariate Cox regression. **(D)** PPI network of the 28 prognostic FRDEGs; the shade indicates the “hub” degree. **(E)** The correlation among the 28 prognostic FRDEGs.

The research flowchart and corresponding online resources were presented in Graphical Abstract. The three cohorts’ basic characteristics are summarized in Supplementary Table 4. Among all 173 FRGs, 127 (74.0%) were FRDEGs between 535 tumor samples and 59 normal samples (80 were upregulated in tumor samples; 47 were downregulated in tumor samples). The heatmap of the FRDEGs is shown in Figure 1A. We performed the univariate Cox regression for the 173 FRGs and extracted 31 prognostic FRGs based on OS in the TCGA cohort (Figure 1B). Twenty-eight overlapping prognostic FRDEGs in FRDEGs and prognostic FRGs were acquired (Figure 1C). We utilized STRING and Cytoscape to detect the intrinsic interactions between these 28 genes. The settings defaulted (network type: full network; active interaction sources: Textmining, Experiments, Database, Co-expression, Neighborhood, Gene Fusion, and Co-occurrence; minimum required interaction score: 0.4). The PPI enrichment *P*-value was 8.09e-07, indicating the network has significantly more interactions than a random protein set. The PPI network (Figure 1D) selected six genes, *KRAS*, *NRAS*, *FLT3*, *SLC7A11*, *TLR4*, *GCLC*, as “hub genes” because their “Degrees” calculated by “cytohubba” were higher than 3, indicating these genes were crucial elements of this biological network. We calculated the Pearson correlation analysis between each two of the 28 prognostic FRDEGs to explore the potential interactions among these genes (Figure 1E) (the cutoff Pearson correlation coefficient was 0.2). The interrelationship between these 28 genes was complicated. For example, *ACSL3* was positively related to seven genes (*KRAS*, *FLT3*, *NRAS*, *VDAC2*, *GCLC*, *PGD*, and *SLC7A11*) and negatively related to *DPP4*. These findings revealed many interactions between the gene pairs, and the regulatory mechanisms of ferroptosis in LUAD were complex.

### Construction of the Prognostic Model

**FIGURE 2:**
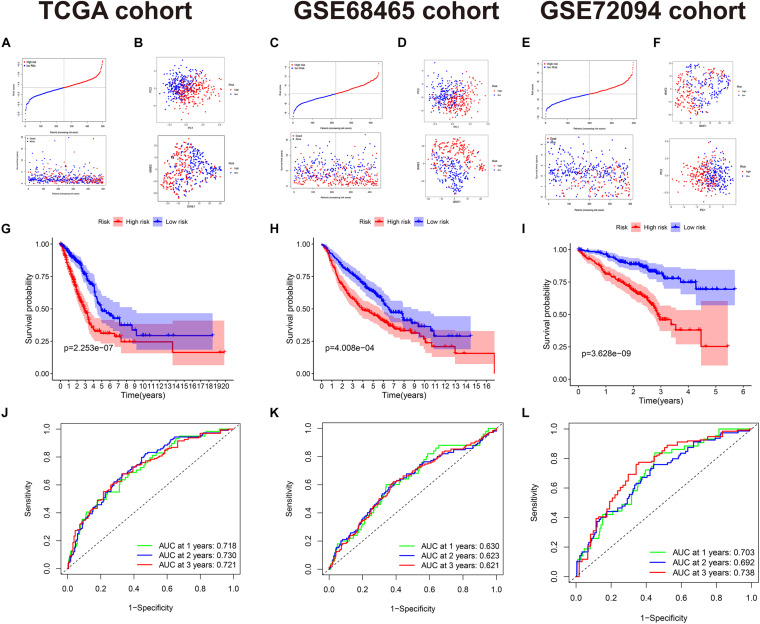
The 15-gene signature in the TCGA, GSE68465, and GSE72094 cohorts. **(A,C,E)** The high- and low-risk groups divided by the median value of risk score and the distribution of the OS and OS status according to the risk score. **(B,D,F)** PCA and t-SNE analyses. PCA and t-SNE indicate the internal diversity between the two groups is obvious. **(G–I)** Survival analysis between the high- and low-risk groups based on OS. **(J–L)** ROC curve of the 15-gene signature model.

Among the 28 FRDEGs, *LINC00336* was excluded because it is a long non-coding RNA. Multivariate Cox regression of the 27 FRDEGs established a 15-gene signature. The risk score was calculated using the formula: Risk score = 0.063*ACSL3* + 0.224*ALOX12B*-0.011 *ALOX15* + 0.155*CISD1* + 0.104*FANCD2*-0.308*FLT3*-0.569 *GLS2* + 0.066*KRAS* + 0.118*PANX1*-0.358*PEBP1* + 0.010*PGD*-0.251*PHKG2* + 0.093*SLC3A2*-0.176*TLR4* + 0.012*VDAC2* {the gene symbol means the expression of the gene [log2(normalized gene expression + 1)]}. The median value of risk score (−1.58) divided the 500 patients into two groups (250 in the high-risk group/250 in the low-risk group) (Figure 2A). The parameter selection of LASSO is presented in Supplementary Figure 1. The basic characteristics of each group are displayed in Supplementary Table 5. The high-risk group contained more male cases and a higher tumor stage. The results of the PCA and t-SNE implied these two groups had diverse trends (Figure 2B). Survival analysis between the two groups is presented in Figure 2G. Notably, the OS in the high-risk group was lower (*P* < 0.001). The ROC curve showed the area under the curve (AUC) reached 0.718, 0.730, and 0.721, respectively, which was acceptable (Figure 2J). The survival analysis of these 15 FRPSGs using GEPIA based on OS is presented in Supplementary Figure 2. *TLR4*, *PHKG2*, *PEBP1*, *GLS2*, *FLT3*, and *ALOX15* were positively associated with the prognosis, and *VDAC2*, *PGD*, *PANX1*, *KRAS*, *ALOX12B*, *ACSL3*, *CISD1*, *FANCD2*, and *SLC3A2* were negatively associated with the prognosis, among which *VDAC2*, *GLS2*, *FLT3*, *TLR4*, *PGD*, *PANX1*, *PEBP1*, *ACSL3*, *CISD1*, *FANCD2*, and *SLC3A2* were of statistical significance. These findings were validated using the Prognoscan database (Supplementary Figure 3). A brief introduction of the 15 FRPSGs is presented in Supplementary Table 6 (Dixon et al., 2012; Kang et al., 2014; Gao et al., 2015; Song et al., 2016; Yang et al., 2016; Yuan et al., 2016; Wenzel et al., 2017; Chen et al., 2019; Magtanong et al., 2019; Su et al., 2019; Wang et al., 2019b). The expression of 15 FRPSGs in different LUAD pathological subtypes is presented in Supplementary Figure 4. The expression of 15 FRPSGs was similar in each pathological subtype, except *FANCD2*. *FANCD2* was lowly expressed in micropapillary carcinoma and highly expressed in solid carcinoma, indicating *FANCD2* could participate in pathological changes in LUAD. We also compared the expression of 15 FRPSGs in different LUAD stages (Supplementary Figure 5). We found that the three ferroptosis suppressor genes (*ACSL3*, *CISD1*, and *FANCD2*) were increased, and two ferroptosis driver genes (*FLT3* and *PHKG2*) were decreased in higher-stage LUAD. However, some ferroptosis driver genes were increased in higher-stage LUAD. We hypothesized that these genes might involve other regulatory pathways of tumor behavior. For example, PGD is highly expressed in stage IV LUAD. In addition, many previous studies have demonstrated that overexpression of PGD could lead to cancer metastasis and poor prognosis through diverse mechanisms (Bechard et al., 2018; Sarfraz et al., 2020).

### Validation on the GSE68465 and GSE72094 Cohorts

The acquired model was applied to the GSE68465 and GSE72094 cohorts. In GSE68465, 442 patients were divided into two groups (-6.88) (Figure 2C). The basic characteristics are displayed in Supplementary Table 5. The high-risk group had more males, more smokers, and a higher T/N stage. PCA and t-SNE analyses also implied these two groups have diverse trends (Figures 2D,F). OS in the high-risk group was still higher (*P* < 0.001) (Figure 2H). AUCs reached 0.630, 0.623, and 0.621 at 1, 2, and 3 years (Figure 2K), which were not very prominent, maybe due to the heterogeneity between the TCGA cohort and the GSE68465 cohort (the mean value of OS was 654.5 days in the TCGA and was 1410 days in the GSE68465). In the GSE72094, 398 patients were divided into two groups (-0.735) (Figure 2E). OS in the high-risk group was higher (*P* < 0.001) (Figure 2I). AUCs reached 0.703, 0.692, and 0.738 at 1, 2, and 3 years, which were very prominent for validation cohorts and almost the same as that in the TCGA cohort, indicating our model is robust and had an excellent predictive ability in similar cohorts [the mean value of OS was 824 days, close to that of the TCGA cohort (654.5 days)] (Figure 2L). We attributed the difference among the median risk score of the TCGA, GSE68465, and GSE72094 cohorts to the diversity between the RNA-seqs and microarrays. The median risk scores of GSE68465 (-0.688) and GSE72094 (-0.735) were similar but far from that of the TCGA cohort (-0.158), which supported our hypothesis.

### Independent Prognostic Analysis

**FIGURE 3:**
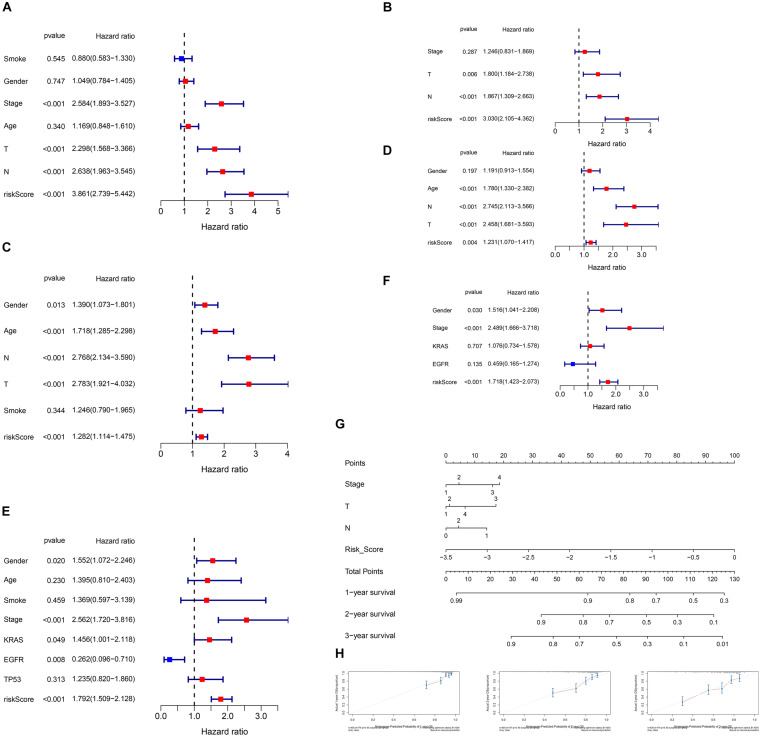
Independent prognostic analysis of the risk score and nomogram with calibration curves of the prognostic factor screened by multivariate Cox regression. **(A)** Univariate Cox regression of the TCGA cohort. **(B)** Multivariate Cox regression of the TCGA cohort. **(C)** Univariate Cox regression of the GSE68465 cohort. **(D)** Multivariate Cox regression of the GSE68465 cohort. Gender, male vs. female; smoke, all smoked vs. never smoked; Stage, high (III/IV) vs. low (I/II); T, high (3/4) vs. low (1/2); N, involved (1/2/3) vs. not involved (0); age, high (>60) vs. low (≤60); risk score, as a continuous variable. **(E)** Univariate Cox regression of the GSE72094 cohort. **(F)** Multivariate Cox regression of the GSE72094 cohort. **(G)** Nomogram of the TCGA cohort [due to only two patients with N3, N3 was considered as NA (missing values)]. **(H)** Calibration curve of the TCGA cohort.

Because of the clinical data difference among the TCGA, GSE68465, and GSE72094 cohorts, the included prognostic factors for OS in the independent prognostic analysis were diverse. In the TCGA cohort, stage, T, N, age, gender, smoking history, and risk score were included. Univariate Cox regression showed stage, T, N, and the risk score are of significance (Figure 3A). Multivariate analysis selected T, N, and the risk score as prognostic factors (Figure 3B). In the GSE68465 cohort, T, N, age, gender, smoking history, and the risk score were included. The uni- and multivariate Cox regression showed that T, N, age, and risk score were statically significant (Figures 3C,D). In the GSE78094 cohort, gender, age, stage, smoking history, *KRAS*, *EGFR*, and *TP53* mutation were included. The uni- and multivariate Cox regression showed that gender, stage, and risk score were statistically significant independent prognostic factors (Figures 3E,F).

### Construction of the Nomogram

We also constructed the nomogram of our 15-gene prognostic signature for clinical reference in the TCGA cohort because RNA-seq is the most utilized gene detection method in the clinic. All the prognostic factors selected by univariate Cox regression of the independent analysis were included in the nomograms. The nomograms and calibration curves are presented in Figures 3G,H. The calibration curve indicates that the fitting and predictive ability of our model were preferable.

### Functional Analyses

**FIGURE 4:**
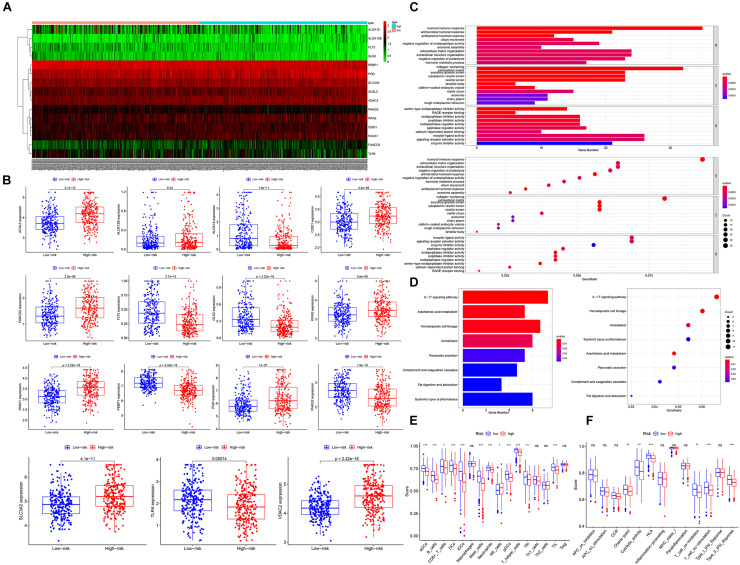
Functional analysis between the high- and low-risk groups. **(A)** The heatmap of differentially expressed genes between the high- and low-risk groups. **(B)** The box plot of comparison of differentially expressed genes in high and low risk. **(C)** The bar plot and bubble plot of GO and KEGG in the TCGA cohort, GSE68465 cohort, and GSE72094 cohort. **(D)** The immune cell infiltrating score and immune function score in the TCGA cohort **(E)** and GSE68465 cohort **(F)**. q-value, adj.*P*-value; ns, not significant; *P < 0.05; **P < 0.01; ***P < 0.001.

Given that ferroptosis is closely related to the malignant progression of various cancer types, we suspected that the ferroptosis level in the high-risk groups was lower than that in the low-risk groups. As shown in Figures 4A,B, according to the Mann–Whitney test (*P* < 0.05), all of the four ferroptosis suppressor genes were significantly upregulated in the high-risk groups, and most ferroptosis driver genes (6/9, 66.7%) were significantly downregulated in the high-risk groups (TCGA cohort). In addition, only three ferroptosis driver genes (*KRAS*, *PANX1*, and *PGD*) were significantly upregulated in the high-risk groups. Furthermore, the ferroptosis suppressor genes were also upregulated in the high-risk groups in the GSE68465 (2/2 suppressor genes were upregulated; 5/9 driver genes were downregulated) and GSE72094 (3/3 suppressor genes were upregulated; 5/8 driver genes were downloaded) cohorts (Supplementary Figures 6A,B). The detailed list of differentially expressed FRPSGs is provided in Supplementary Table 7. Therefore, we suggested that the ferroptosis level was lower in the high-risk groups. To explore the potential biological processes influenced by the ferroptosis process, we utilized the R package “limma” to extract DEGs between the high- and low-risk groups in the three cohorts. A total of 391 DEGs (147 upregulated and 244 downregulated in the high-risk group) in the TCGA cohort, 438 DEGs (285 upregulated and 153 downregulated in the high-risk group) in GSE68465, and 377 DEGs (116 upregulated and 261 downregulated in the high-risk group) between the high- and low-risk groups were extracted. Notably, 20 genes were upregulated and 20 genes were downregulated in the high-risk groups in all three cohorts. A detailed list of DEGs is provided in Supplementary Table 8. The GO and KEGG pathway enrichment indicated that the three cohorts’ DEGs were massively enriched on cell metabolism and cell cycle, which revealed the association between ferroptosis and these fundamental biological processes (Figures 4C,D and Supplementary Figures 6E–H). Given that ferroptosis is associated with tumor immunity and can influence tumor immunotherapy outcomes, it is noteworthy that several immune pathways were enriched. For example, in the TCGA cohort, the enrichment appeared on immune-related pathways (humoral immune response, etc.) in the GO database (Figure 4C) and three immune-related pathways (IL-17 signaling pathway, etc.) in the KEGG database (Figure 4D). These enriched immune-related pathways indicated that the ferroptosis process involves the development of tumor immune evasion through changing TME. Similarly, in the GSE68465 and GSE72094 cohorts, many immune-related pathways were enriched (Supplementary Figures 6E–H).

To elucidate the impact of ferroptosis on TME, we performed ssGSEA to compare the two groups’ immune scores. The results showed that dendritic cells (DCs), activated DCs (aDCs), B cells, CD8 + T cells, iDCs, neutrophils, pDCs, T helper cells, Tfh, mast cells, and TIL were significantly reduced in the high-risk group (TCGA) (Figure 4E). As for immune function pathways, HLA, T cell coinhibition, checkpoint, cytolytic activity, T cell costimulation, and type II interferon (IFN) response were reduced (TCGA) (Figure 4F). These findings strongly suggested that the low-risk group patients’ TME had a more potent antitumor effect than the high-risk group, indicating immunotherapy outcomes were better in high-risk group patients, which was consistent with previous reports (Xu et al., 2021).

### Pan-Cancer Analysis

**FIGURE 5:**
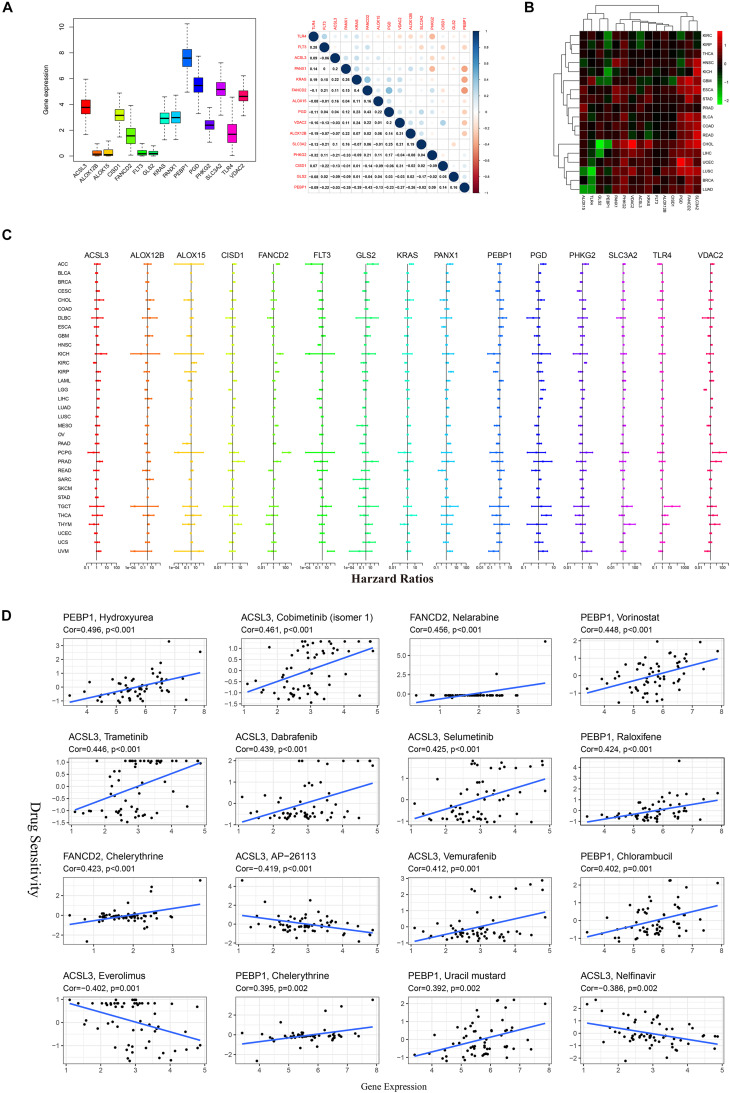
Pan-cancer analysis of 15 FRPSGs. **(A)** The expression level of FRPSGs and correlation of gene expression among FRPSGs. **(B)** Heatmap of FRPSGs between tumor and normal samples in each cancer type. **(C)** Forest plots for hazard ratios of FRPSGs for each cancer type. **(D)** Scatter plots for the association between FRPSG expression and drug sensitivity (top 16 ranked by *P*-value).

The expression of 15 FRPSGs in pan-cancer was different (Figure 5A). The expression of *ALOX12B*, *ALOX15*, *FLT3*, and *GLS2* was deficient, and the expression of *PEBP1* was the highest, indicating *PEBP1* could be a promising treatment target. Spearman correlation showed that *PEBP1* and *FANCD2* had the highest correlation. There were some potential interactions between these two genes. Gene differential expression analysis showed that the 15 FRPSGs were differently expressed in tumor tissues compared with corresponding normal tissues. It is noteworthy that the ferroptosis suppressor genes, *SLC3A2*, *FANCD2*, *CISD1*, and *ACSL3*, were upregulated in most cancer types, indicating that ferroptosis was generally inhibited in cancers (Figure 5B). As for survival analysis, the same gene had different prognostic significance in distinct cancer types. For example, *VDAC2* was negatively associated with OS in low-grade glioma (LGG) and positively in pheochromocytoma and paraganglioma (PCPG) (Figure 5C). It is reported that ferroptosis is a critical regulator in drug resistance. Here, we utilized the NCI-60 database to explore the association between FRPSGs and drug sensitivity using Pearson correlation. The top 16 gene–drug pairs ranked by Pearson correlation coefficient are displayed in Figure 5D. The whole list of the drug sensitivity analysis results is in Supplementary Table 9. We found that the ferroptosis driver gene, *PEBP1*, was positively related to chemotherapy sensitivity, consistent with previous reports. Interestingly, the ferroptosis suppressor gene, *ACSL3*, was negatively related to three drug sensitivity, among which AP-26113 is a potent and selective ALK and ROS1 inhibitor for lymphoma, everolimus is an mechanistic target of rapamycin (mTOR) kinase inhibitor for renal cell cancer and some other tumors, and nelfinavir is a protease inhibitor for HIV infection. These findings demonstrated that ferroptosis was involved in some tumor-targeted therapy.

**FIGURE 6:**
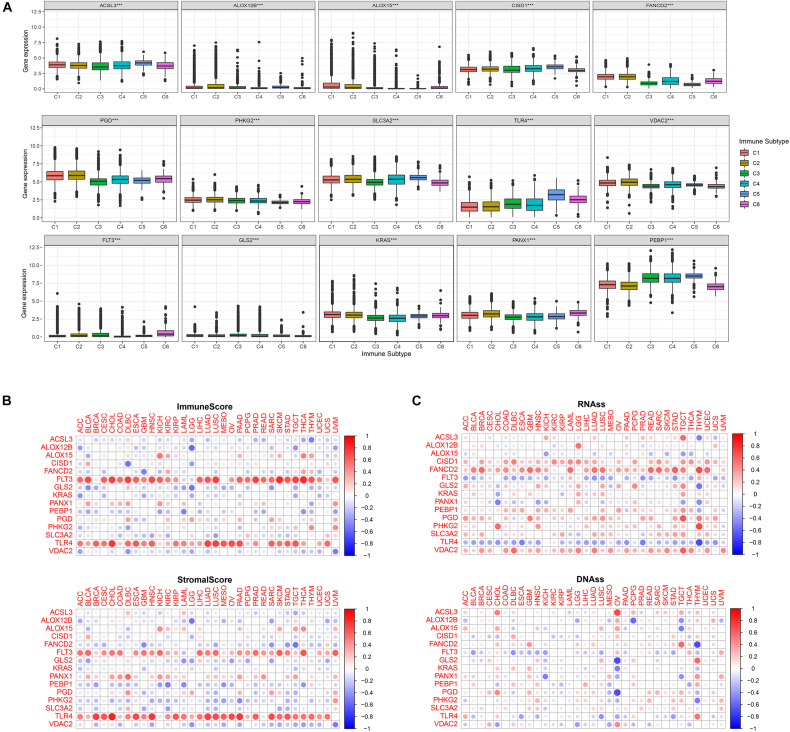
Immune subtype analysis, TME analysis, and cancer stemness features. **(A)** The association between FRPSGs and immune subtype for each cancer type. **(B)** The association between ESTIMATE immune score, stromal score, and FRPSGs expression. **(C)** The association between cancer stemness features and FRPSGs expression based on both mRNA expression (RNAss) and DNA methylation patterns (DNAss).

Immune analysis of the 15 FRPSGs was performed. Six immune subtypes were defined as C1 (wound healing), C2 (INF-r dominant), C3 (inflammatory), C4 (lymphocyte depleted), C5 (immunologically quiet), and C6 (TGFβ dominant). As shown in Figure 6A, all the 15 FRPSGs expressed differently in diverse immune subtypes (*P* < 0.001) for each cancer type. Moreover, TME analysis showed the 15 FRPSGs were related to immune and stromal cell infiltration in most cancer types (Figure 6B). These findings were consistent with functional analysis, which further proved that the ferroptosis process involves the change of TME and tumor immunity. Finally, we performed the cancer stemness feature analysis. A stem cell approximative appearance can be gained via cancer growth progress; simultaneously, a differentiated phenotype is eliminated. Based on RNAss (mRNA expression) and DNAss (DNA-methylation pattern), the four ferroptosis suppressor genes, *ACSL3*, *CISD1*, *FANCD2*, and *SLC3A2*, increased the tumor’s stem cell-like features, which indicated that the ferroptosis could cause tumor heterogeneity, leading to tumor progression in most cancer type (Figure 6C). Specifically, in LUAD, the four genes were positively associated with RNAss and DNAss (*P* < 0.05), especially *FANCD2* (Supplementary Figure 7). The Spearman correlation between *FANCD2* expression and RNAss reached 0.56 (*P* < 2.2e-16), indicating *FANCD2* plays a critical role in promoting tumor heterogeneity. We further validated these findings by exploring the association between the four ferroptosis suppressor genes with two previously discovered LUAD stemness cell biomarkers, CD133 and CD44. As shown in Supplementary Figure 8, the results show that the four ferroptosis suppressor genes were all positively associated with CD133 and CD44 (*P* < 0.05), which were consistent with the results of cancer stemness feature analysis. In summary, we identified that the four ferroptosis suppressor genes, *ACSL3*, *CISD1*, *FANCD2*, and *SLC3A2*, could increase the cancer stemness, indicating that the lower the ferroptosis level in lung cancer cells, the higher the tumor heterogeneity.

### Somatic Mutation and CNV Analysis

**FIGURE 7:**
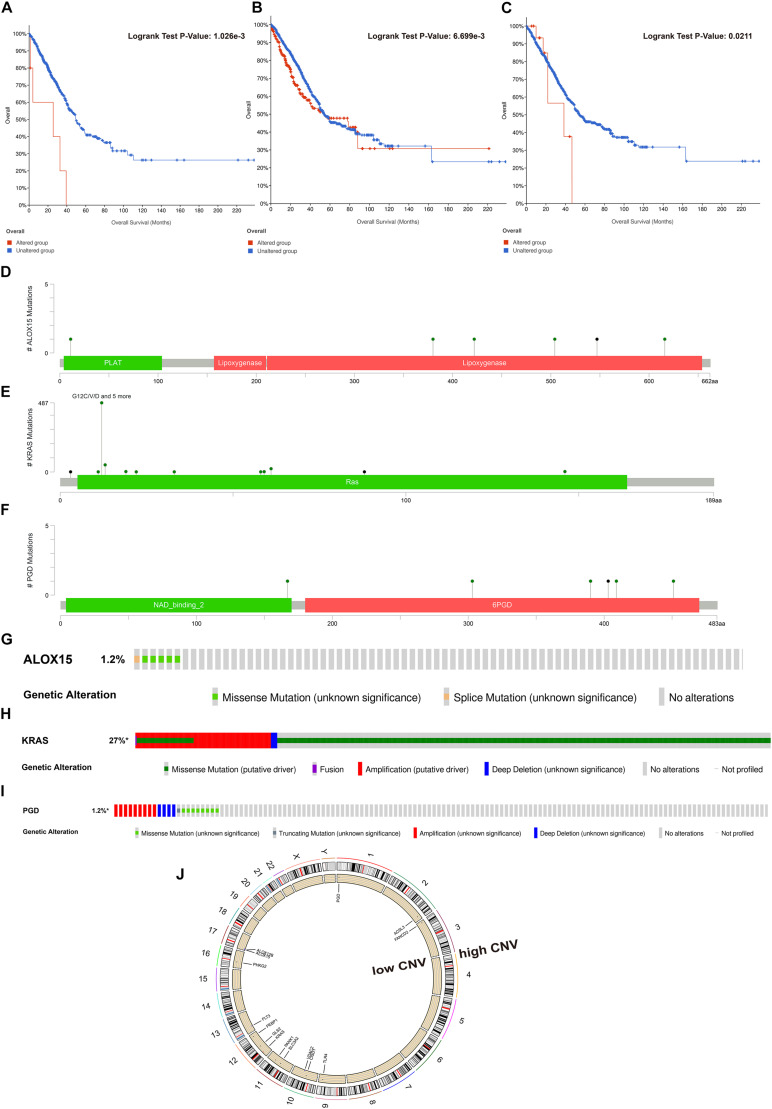
Somatic mutation and CNV analysis of *ALOX15*, *KRAS*, and *PGD* in LUAD. Survival analysis between the altered group and unaltered group based on OS for *ALOX15* **(A)**, *KRAS* **(B)**, and *PGD* **(C)**. The alteration sites of each gene on the coding protein domain for *ALOX15* **(D)**, *KRAS* **(E)**, and *PGD* **(F)**. The alteration rate and type of *ALOX15* **(G)**, *KRAS* **(H)**, and *PGD* **(I)**. **(J)** The location on chromosomes and most common CNV status of the 15 FRPSGs.

**FIGURE 8:**
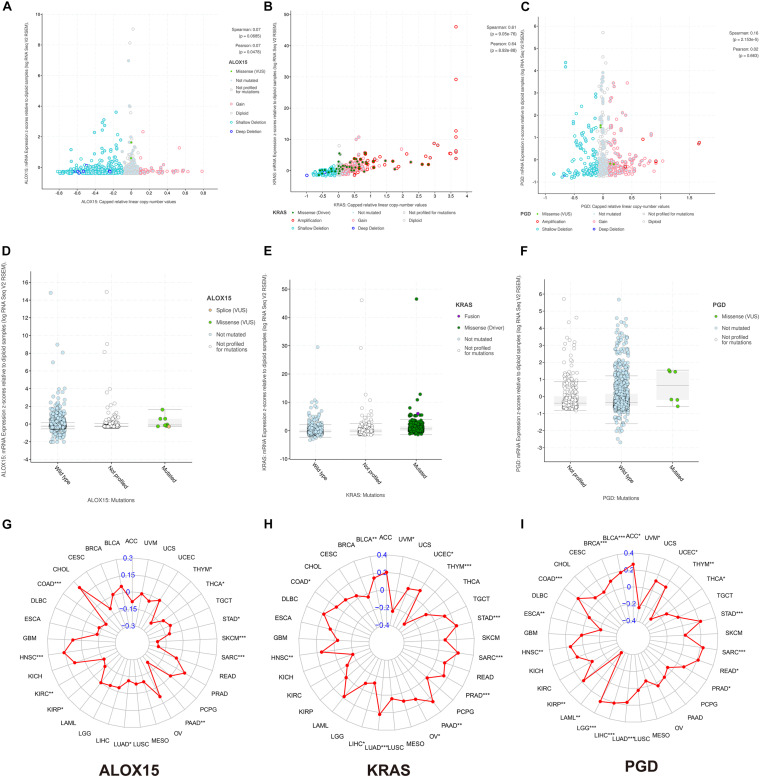
The association between gene expression and alteration status in adenocarcinoma using cBioPortal and TMB of *ALOX15*, *KRAS*, and *PGD* in pan-cancer. The association between gene expression and CNV for *ALOX15* **(A)**, *KRAS* **(B)**, and *PGD* **(C)**. Comparison of gene expression between wild type and mutation type for *ALOX15* **(D)**, *KRAS* **(E)**, and *PGD* **(F)**. The association between gene expression and TMB status in pan-cancer for *ALOX15* **(G)**, *KRAS* **(H)**, and *PGD* **(I)**. *P < 0.05; **P < 0.01; ***P < 0.001.

To fundamentally understand the ferroptosis process during tumor development, we performed mutation analysis of the 15 FRPSGs. The location on chromosome and CNV status of the 15 FRPSGs are presented in Figure 7J. Four ferroptosis driver genes *FLT3*, *ALOX12B*, *ALOX15*, and *VDAC2*, had a low CNV, and four ferroptosis suppressor genes *ACSL3*, *CISD1*, *FANCD2*, and *SLC3A2*, had a high CNV, indicating that ferroptosis was inhibited in tumor development. Besides, the generally accepted oncogene *KRAS* had a high CNV. Among the 15 FRPSGs, the alteration status of three genes, *ALOX15*, *KRAS*, and *PGD*, was significantly related to OS analysis (Figures 7A–C, *P* = 1.026e-3, 6.699e-3, 0.0211), with an alteration rate of 1.2, 27, and 1.2% (Figures 7G–I). Besides, the alterations of *AOX15*, *KRAS*, and *PGD* mainly focused on domain lipoxygenase, Ras, and 6PGD, indicating that these domains played a crucial role in ferroptosis and tumor progression (Figures 7D–F). The alteration type of the three genes is shown in Figures 7G–I. Missense mutation was the most common mutation type. Splice mutation occurred in *ALOX15* alteration, fusion mutation occurred in *KRAS* alteration, and truncating mutation occurred in *PGD* mutation. The rate of these three mutation types was low. CNV was rare in *ALOX15* alteration but common in *KRAS* and *PGD* alteration, mainly consisting of amplification and deep deletion. Figures 8A–F showed the association between gene expression and alteration status in adenocarcinoma using cBioPortal and TMB of *ALOX15*, *KRAS*, and *PGD* in pan-cancer. The association between gene expression and CNV for *KRAS* and *PGD* was statistically significant (by Spearman correlation, *P* = 9.05e-76, 2.153e-5) with correlations of 0.61 and 0.16 (Figures 8B,C). Comparison of gene expression between wild type and mutation type for *ALOX15*, *KRAS*, and *PGD* indicated mutation of these three genes could increase their expression (Figures 8D–F). The most common mutation type was missense for three genes. Besides, splice mutation occurred in *ALOX15*, and fusion mutation occurred in *KRAS*. The association between gene expression and TMB status in pan-cancer for *ALOX15*, *KRAS*, and *PGD* is shown in Figures 8G–I. The expression of *KRAS* and *PGD* was positively associated with TMB in LUAD (*P* < 0.001), and the Spearman correlation reached around 0.2, indicating that *KRAS* and *PGD* played a crucial in maintaining genomic integrity. Given that TMB is positively related to immune therapy response rate, we suppose that *KRAS* and *PGD* could serve as novel biomarkers for predicting immunotherapy response rate.

## Discussion

Among the 15 FRPSGs, most ferroptosis driver genes were associated with a better prognosis (6/11, 54.5%), and all the ferroptosis suppressor genes (4/4, 100%) were associated with a worse prognosis, which indicates that the ferroptosis process in LUAD inhibits the tumor development as previously reported (Stockwell et al., 2017; Liang et al., 2019). We suggested these 10 genes (*TLR4*, *PHKG2*, *PEBP1*, *GLS2*, *FLT3*, *ALOX15*, *ACSL3*, *CISD1*, *FANCD2*, and *SLC3A2*) could be potential targets for further research because their biological functions in ferroptosis were consistent with their prognostic significance.

Lipid metabolism, iron metabolism, (anti)oxidative metabolism, and energy metabolism are the main regulatory pathways of ferroptosis. In our signature, lipid metabolism involves *ACSL3*, *ALOX15*, *ALOX12B*, and *PEBP1*. ACSL3 is a principal modulator of ferroptosis by activating the exogenous monounsaturated fatty acids, which subsequently replace polyunsaturated fatty acids (PUFAs) and block lipid ROS accumulation (Tang et al., 2018; Magtanong et al., 2019). Lipoxygenase (ALOX) is the critical enzyme for lipid peroxidation and ferroptosis through the arachidonic acid (AA) oxygenation. ALOX15 and the scaffold protein PEBP1 form a complex, converting PUFAs (mainly AA) to 15-HPETEs (hydroperoxyeicosatetraenoic acids), and this leads to ferroptosis in several diseases (Anthonymuthu et al., 2018; Zhao et al., 2020). In particular, ALOX15 has long been regarded as a critical mediator and thus a prominent indicator in the seton session of lung cancer (Li et al., 2019a). ALOX12B, R-type of ALOX12 catalyzed by ALOX12, is necessary for p53-mediated antitumor effect via ferroptosis (Mashima and Okuyama, 2015). Jiang et al. (2020) have demonstrated that ALOX12B promotes cervical cancer progression through modulating the PI3K/ERK1 signaling pathway. ALOX12B has a negative relation to the prognosis of LUAD in our study, which indicates that ALOX12B may be involved in some other tumor regulatory mechanisms (Jiang et al., 2020).

(Anti)oxidant metabolism involves *SLC3A2*, *VDAC2*, *GLS2*, and *FLT3*, within which SLC3A2 is the heavy chain subunit of system xc-cystine/glutamate antiporter, maintaining the steady state of redox (Koppula et al., 2020). It is reported that inhibition of SLC3A2 promotes ferroptosis in both tumor cells and normal cells (Koppula et al., 2020; Liu et al., 2020). VDAC2 can interact with Mcl-1, an antiapoptotic member of the Bcl-2 family frequently upregulated in NSCLC. In A549 cells, a decrease in Mcl-1 expression level or application of VDAC-based peptides inhibits Ca (2+) flow into the mitochondrial matrix, leading to the limitation of ROS generation (Huang et al., 2014). GLS2 plays a critical in glutaminolysis, which can provide intracellular glutamate for GSH synthesis. Zhu et al. (2017) knocked down *GLS2* in mouse embryonic fibroblasts and found that the ferroptosis is reduced. Jennis et al. (2016) explored that GLS2 may act as a transcriptional target of TP53 and promote TP53-dependent ferroptosis. FLT3, a receptor tyrosine kinase, was reported as a critical mediator in the process of glutamate oxidative stress-induced cell death. Although we have no clear understanding of what role FLT3 plays in lung cancer until today, FLT3-ligand, commonly regarded as a hematopoietic stimulator, can be used in treating lung infection via functioning in lung immune cell populations (Dessein et al., 2020).

PGD involves energy metabolism such that it functions in the pentose phosphate pathway (PPP). Although the association between energy metabolism and ferroptosis remains puzzling, Dubreuil et al. (2020) have recently discovered that disrupting PPP by PGD knockout inhibits ROS accumulation. Besides, many previous studies have demonstrated that overexpression of PGD could lead to cancer metastasis and poor prognosis (Bechard et al., 2018; Sarfraz et al., 2020). In our study, the alteration rate of *PGD* was more than 1.2% and was related to a significantly poor prognosis. It is noteworthy that the CNV was relatively common in *PGD* alteration. Shallow deletion, amplification, and deep deletion occurred, and the gene expression was significantly related to CNV. More research is needed to explore the relationship between PGD’s CNV and ferroptosis.

The remaining three FRPSGs, *TLR4*, *FANCD2*, and *KRAS*, serve as specific mediators. TLR4 is a toll-like receptor responsible for activating the innate immune system and has been confirmed to be associated with inflammation, autophagy, cell migration, and adhesion/metastasis in NSCLC (Kim et al., 2020). Li et al. (2019b) constructed a heart ischemia–reperfusion model. They demonstrated that the TLR4/TRIF/type I IFN signaling pathway impacts recruiting neutrophils to injured myocardium in the ferroptosis process (Li et al., 2019b). FANCD2, a nuclear protein involved in DNA reparation, prevents bone marrow stromal cells from ferroptosis via iron accumulation and lipid peroxidation inhibition, which is fulfilled by maintaining DNA stability when treated with ferroptosis inducer such as erastin (Song et al., 2016). FANCD2 has been verified that its exhaustion would increase cancer cell proliferation in the knockout experiment on the PDX model. The overexpression of FANCD2 would bring an increased risk of the incidence of lung cancer (Wang et al., 2015; Yao et al., 2015). KRAS, one of the most frequently mutated oncogenes in human cancer, is a signal transducer protein in cell proliferation. It has been reported that erastin has exhibited greater lethality in *KRAS*-mutated cancer cells (Yagoda et al., 2007). On the contrary, Schott et al. demonstrated that *KRAS* mutation leads to increased resistance to ferroptosis. Therefore, the exact role of *KRAS* in ferroptosis remains elusive. In this study, we considered *KRAS* as a ferroptosis driver gene according to the FerrDb database. The alteration rate of *KRAS* reached 27% in LUAD patients. Besides, the high CNV status of *KRAS* means it is easy to detect at an early stage. ALL implies KRAS could be the best ferroptosis-based treatment target and biomarker.

Ferroptosis is a highly complicated cellular process involving lipid, iron, and cysteine metabolism (Stockwell et al., 2017). Discussion on specific genes cannot explain the complex mechanisms involved explicitly. Therefore, we performed a functional analysis. The results showed that the tumor cellular fundamental biological behavior within the high- and low-risk groups differ. Previous studies have demonstrated that many oncogenes and antioncogenes act as ferroptosis regulators in cancers. For example, TP53 can suppress SLC7A11 expression and promote SAT1 (spermidine/spermine N1-acetyltransferase 1), and GLS2 expression to promote ferroptosis. It also can inhibit DPP4 (dipeptidyl peptidase-4) and promote CDKN1A/p21 (cyclin-dependent kinase inhibitor 1A) expression to inhibit the ferroptosis (Xie et al., 2017; Kang et al., 2019). However, the systematic biochemical process and regulation of ferroptosis are unclear. Friedmann Angeli et al. (2019) reviewed current literature and proposed a GPX4 center view depicting the principal metabolic determinants’ modulation controlling the cancer growth and persistence. As for the immune function analysis, it is recently reported that ferroptosis cancer cells also release “find me” signals to recruit antigen-presenting cells, initiating the innate immune response. Unlike apoptosis, potential signals for ferroptosis are AA oxidation products. Therefore, ALOXs also regulate immunity (Friedmann Angeli et al., 2019; Stockwell and Jiang, 2019). The reduction in DCs and HLA, two essential components for antigen-presenting, indicates that the TME in high-risk groups can inhibit signal transmission. Impaired type II IFN response also indicates the innate immunity in high-risk groups is compromised. The function of mast cells and natural killer (NK) cells in LUAD TME remains complicated and elusive. Recent studies have demonstrated that mast cells and NK cells improve the prognosis of LUAD patients (Ko et al., 2017; Aktas et al., 2018).

The pan-cancer analysis comprehensively analyzed these 15 genes. First, the expression and survival analysis indicate that the heterogeneity of 15 FRPSGs is evident among all the 33 cancer types. However, ferroptosis suppressor genes were downregulated in most cancer types, consistent with previous speculations (Sun et al., 2016; Roh et al., 2017; Seibt et al., 2019). Second, drug sensitivity analysis proved that ferroptosis is closely related to chemotherapy resistance. Interestingly, our findings also provide a new perspective that ferroptosis may involve some tumor-targeted therapy resistance. Third, immune subtype and TME analysis proved that these FRPSGs take part in the immune response, TME infiltration. Finally, we found that ferroptosis could contribute to cancer stemness based on cancer stemness indexes and previously discovered biomarkers, providing novel insights into ferroptosis.

Before us, FRG signatures had been established and applied for LUAD in recent research (Gao et al., 2021; Sun et al., 2021). Even so, our signature has better robustness with better predictive ability, providing more solid clinical reference. Our AUCs of the ROC curve were 0.718, 0.730, and 0.721 for 1, 2, and 3 years, respectively, while Sun’s signature AUCs were merely 0.625, 0.588, and 0.593 for 2, 3, and 5 years, respectively; Gao’s AUCs were 0.678, 0.698, and 0.697 for 1, 2, and 3 years, respectively, in the TCGA-LUAD cohort. Moreover, we emphasized the analysis of FRPSGs, trying to elucidate the potential regulatory role of these genes through an integrative multi-omics study, including expression, prognosis, cancer stemness, drug sensitivity analysis, and mutation analysis.

There are also several limitations to our study. First, the GSE68465/GSE72094 cohorts and TCGA cohort are heterologous, especially the OS of GSE68465 (654.5 days in TCGA and 1410 days in GSE68465). Besides, the data quality and preprocessing of the two data resources (RNA-seq and microarray) were different, which lead to different data processing methods and different cutoff values. However, we could not find an appropriate RNA-seq validation cohort currently. Second, the clinical significance of our model needs to be further evaluated through prospective data in the real world. Third, experimental evidence was not acquired to prove our conclusion, including drug sensitivity analysis and cancer stemness analysis.

## Data Availability Statement

The original contributions presented in the study are included in the article/Supplementary Material, further inquiries can be directed to the corresponding author.

## Author Contributions

ZR and MH performed the data analysis and wrote the first draft. ZW and JG revised the manuscript and carried out data collection. XZ and GZ revised the manuscript and tables. HZ designed and supervised the study. All authors contributed to the article and approved the submitted version.

## Conflict of Interest

The authors declare that the research was conducted in the absence of any commercial or financial relationships that could be construed as a potential conflict of interest.

## Publisher’s Note

All claims expressed in this article are solely those of the authors and do not necessarily represent those of their affiliated organizations, or those of the publisher, the editors and the reviewers. Any product that may be evaluated in this article, or claim that may be made by its manufacturer, is not guaranteed or endorsed by the publisher.

## Footnotes

1 https://portal.gdc.cancer.gov/repository, November 5, 2020

2 https://www.ncbi.nlm.nih.gov/geo/query/acc.cgi?acc=GSE68465, December 27, 2020

3 https://www.ncbi.nlm.nih.gov/geo/query/acc.cgi?acc=GSE72094, April 27, 2021

4
http://www.zhounan.org/ferrdb/index.html

5 https://xenabrowser.net/datapages/, December 24, 2020

6 https://discover.nci.nih.gov/cellminer/, April 24, 2021

7
http://bioinformatics.mdanderson.org/estimate/

8
https://www.cbioportal.org/
