# Supplementary figures and images for: Ferroptosis-Related Genes in Lung Adenocarcinoma: Prognostic Signature and Immune, Drug Resistance, Mutation Analysis
Source: Front Genet. 2021 Aug 9;12:672904. doi: 10.3389/fgene.2021.672904 (PMC8381737; doi:10.3389/fgene.2021.672904)

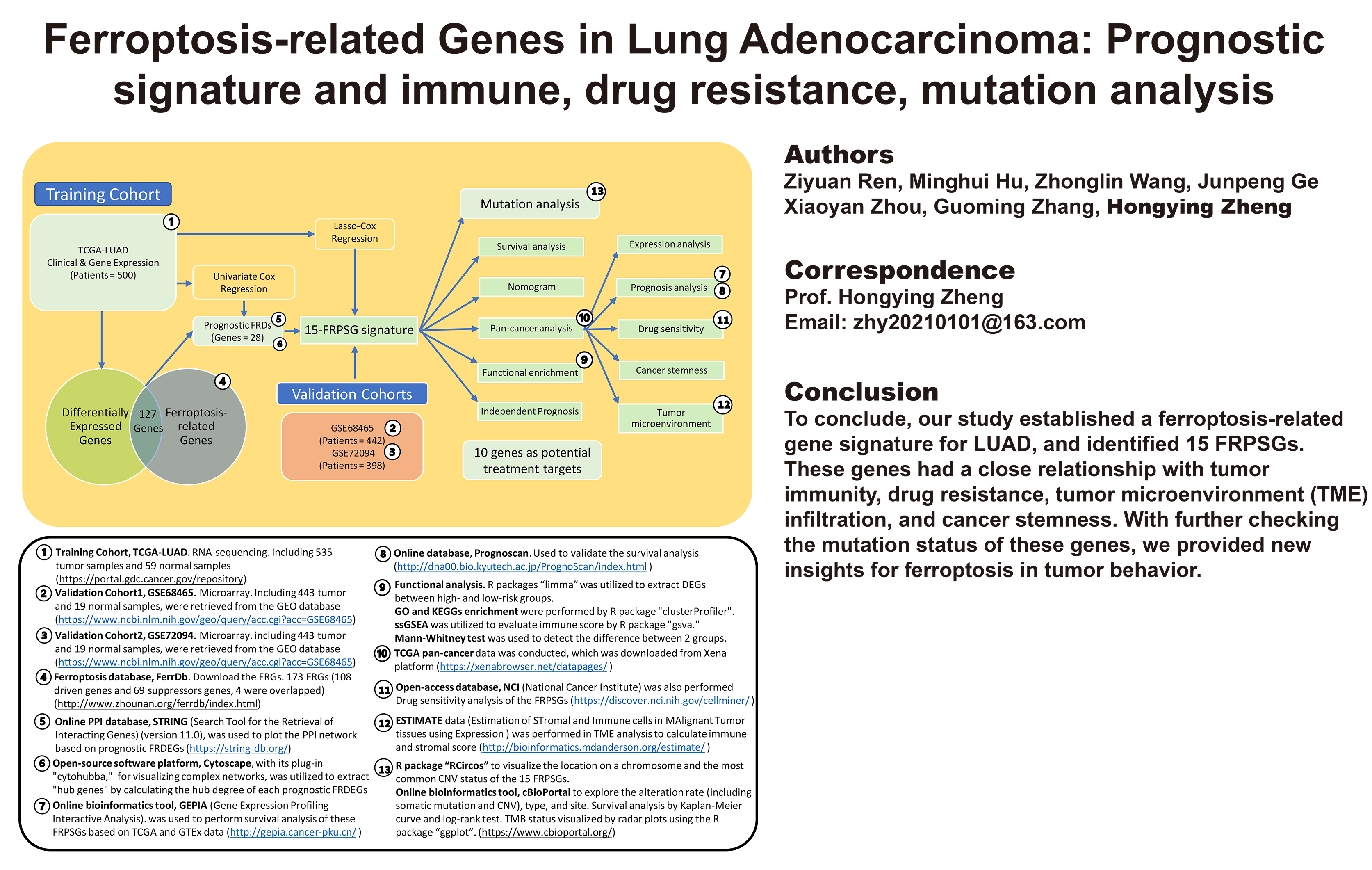

Supplement: Supplementary file 1 [file Image_1.TIF]

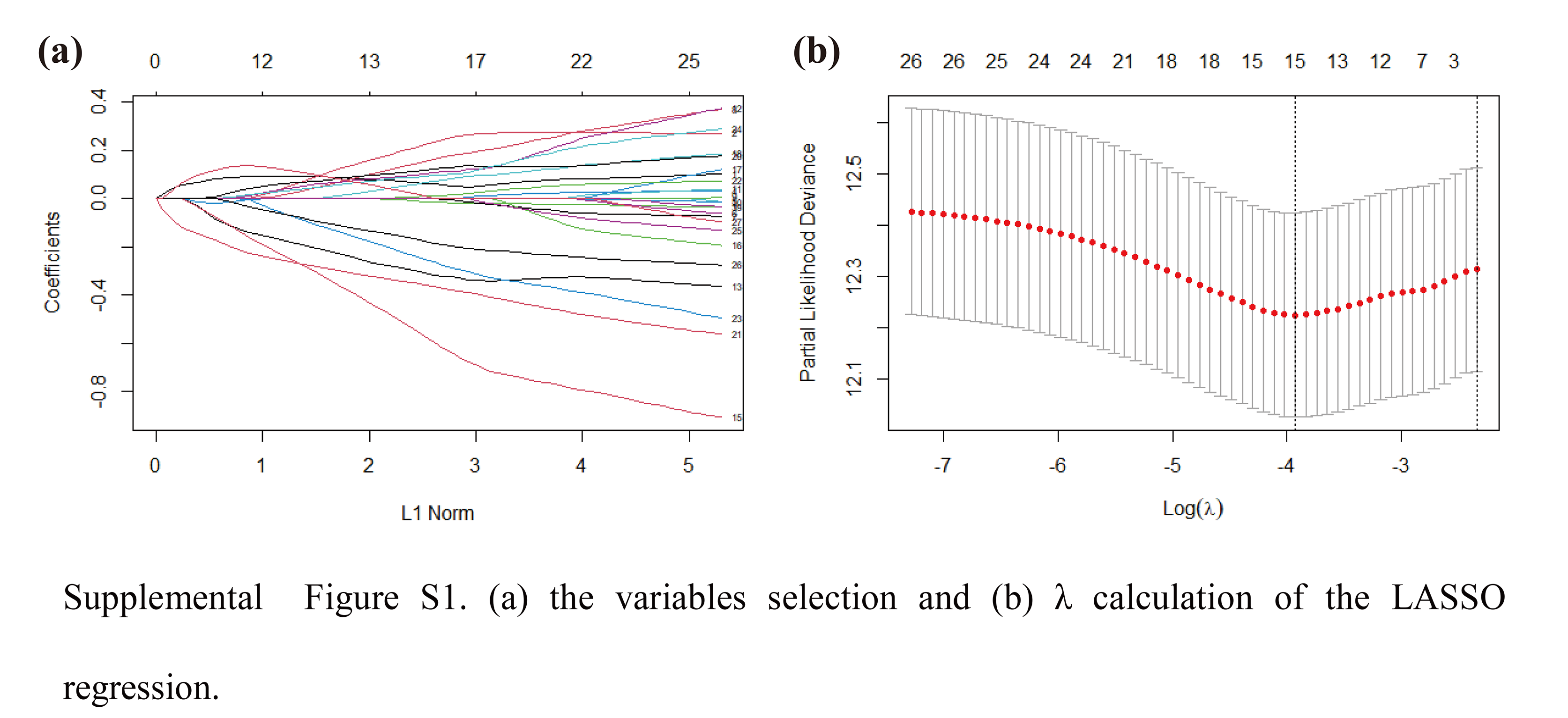

Supplement: Supplementary file 2 [file Image_2.TIF]

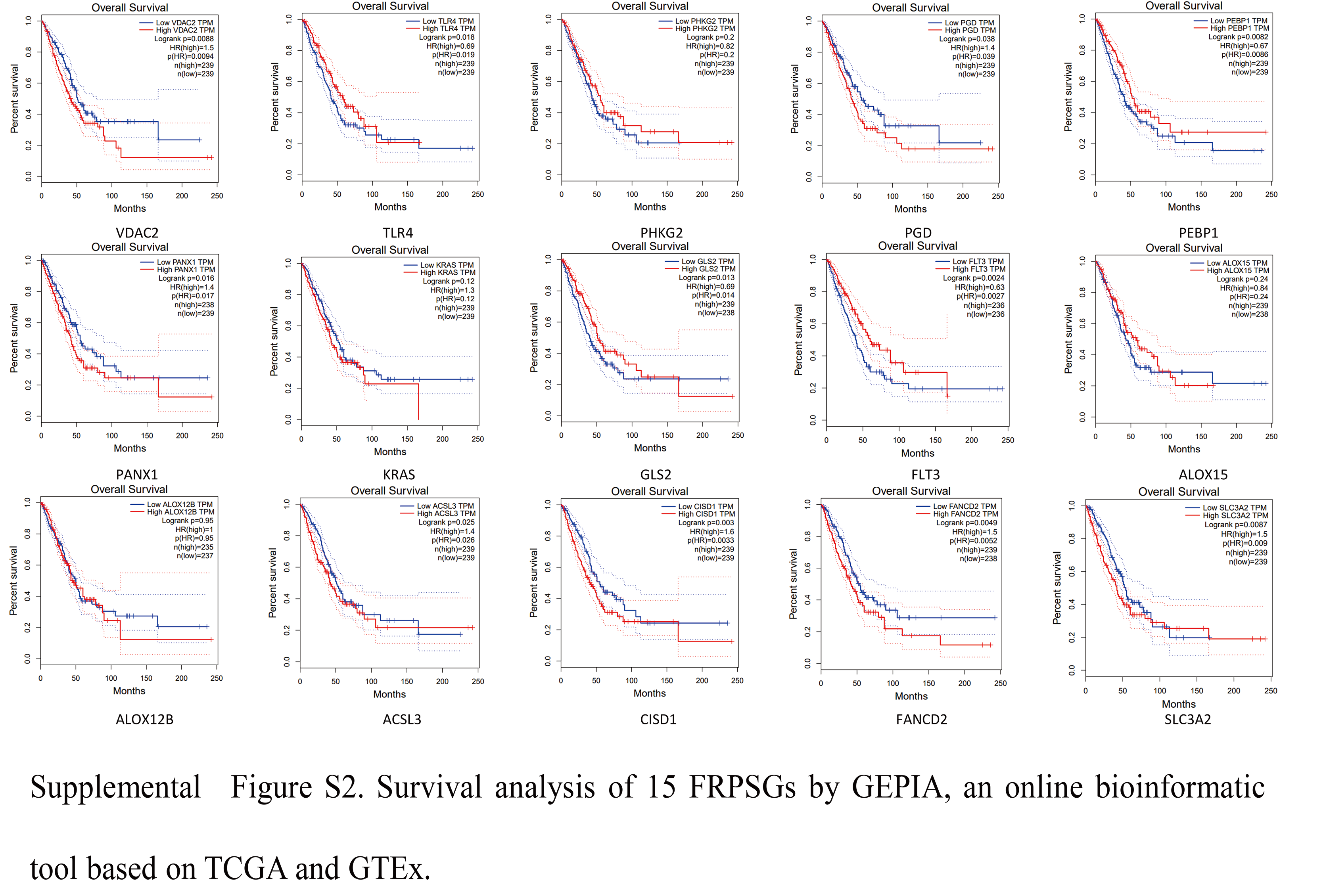

Supplement: Supplementary file 3 [file Image_3.TIF]

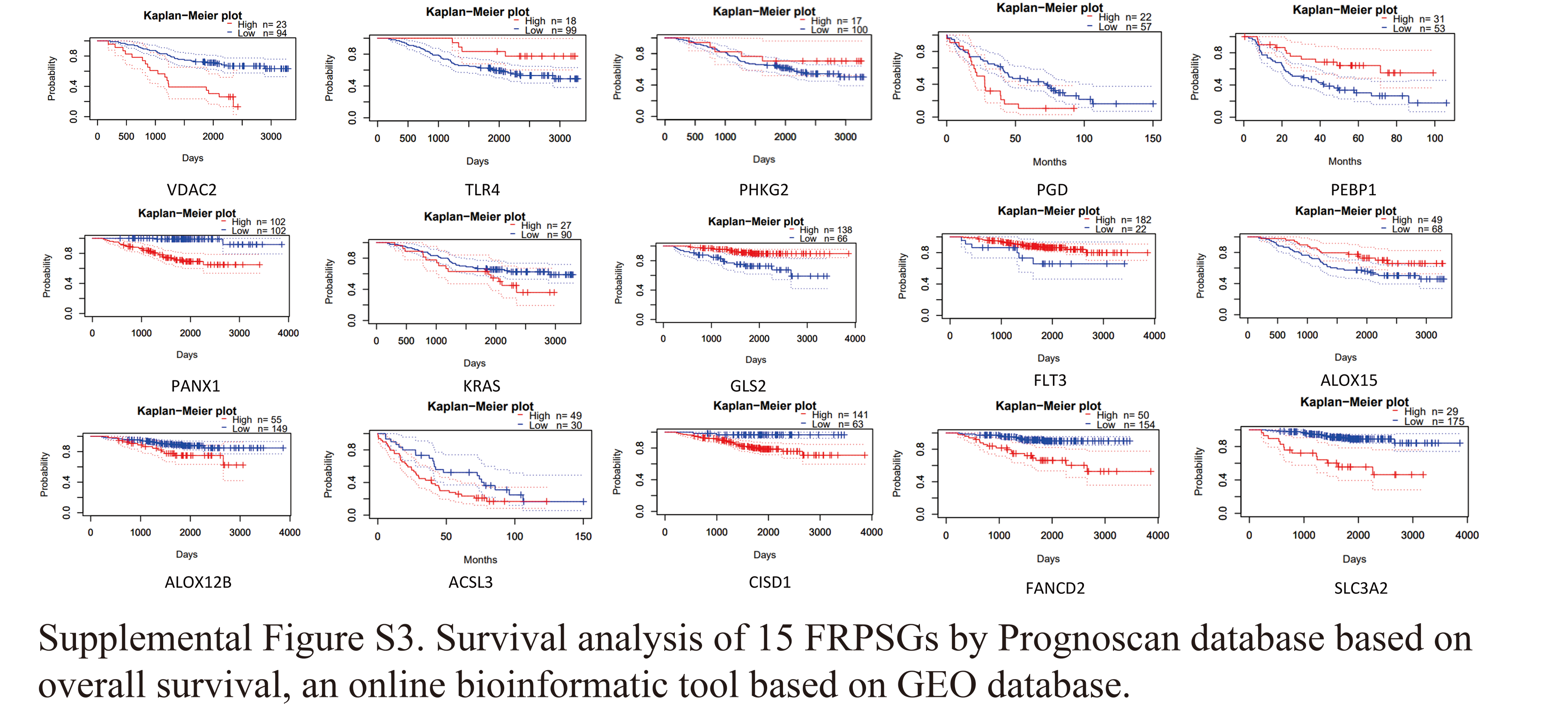

Supplement: Supplementary file 4 [file Image_4.TIF]

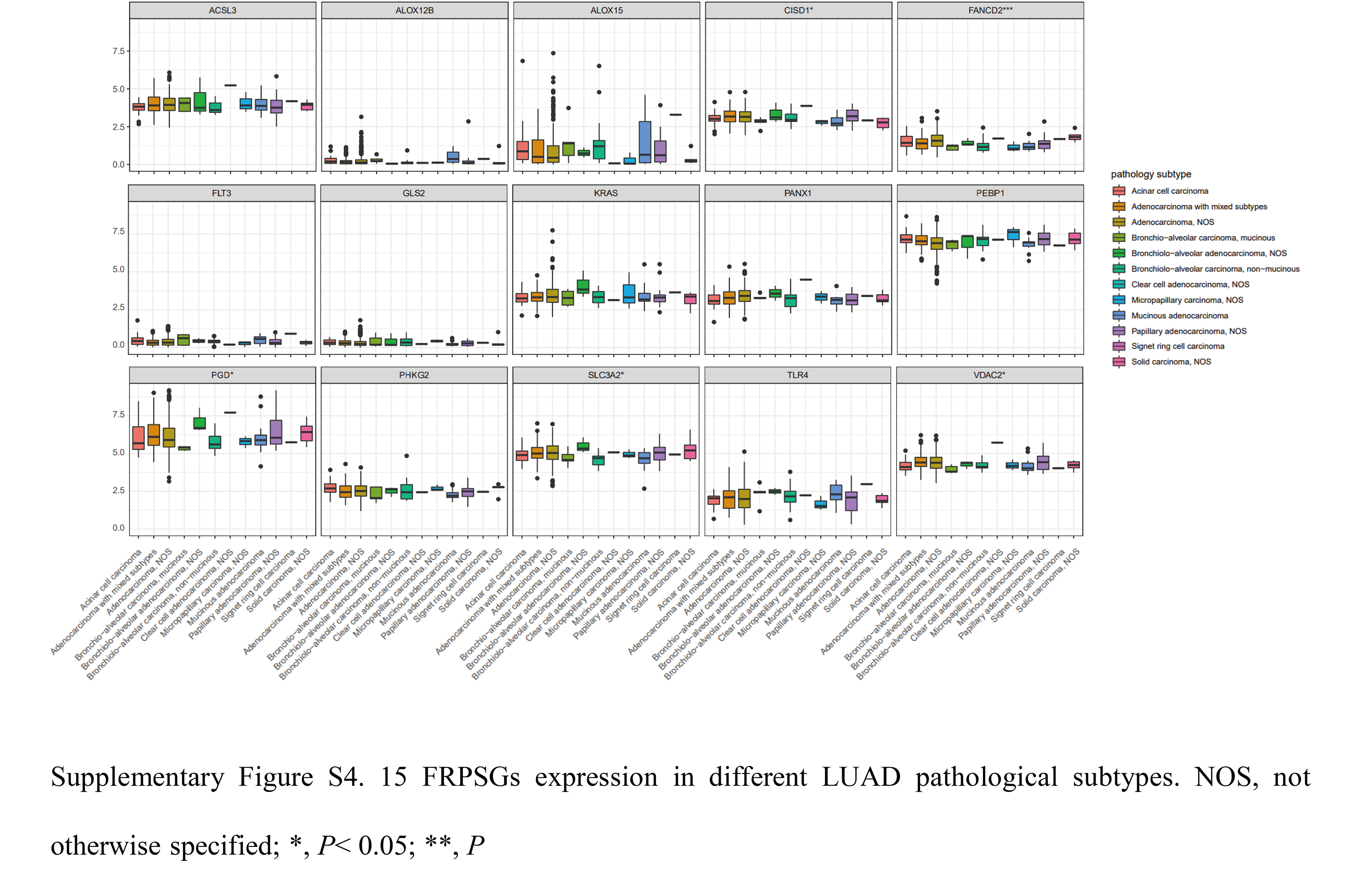

Supplement: Supplementary file 5 [file Image_5.TIF]

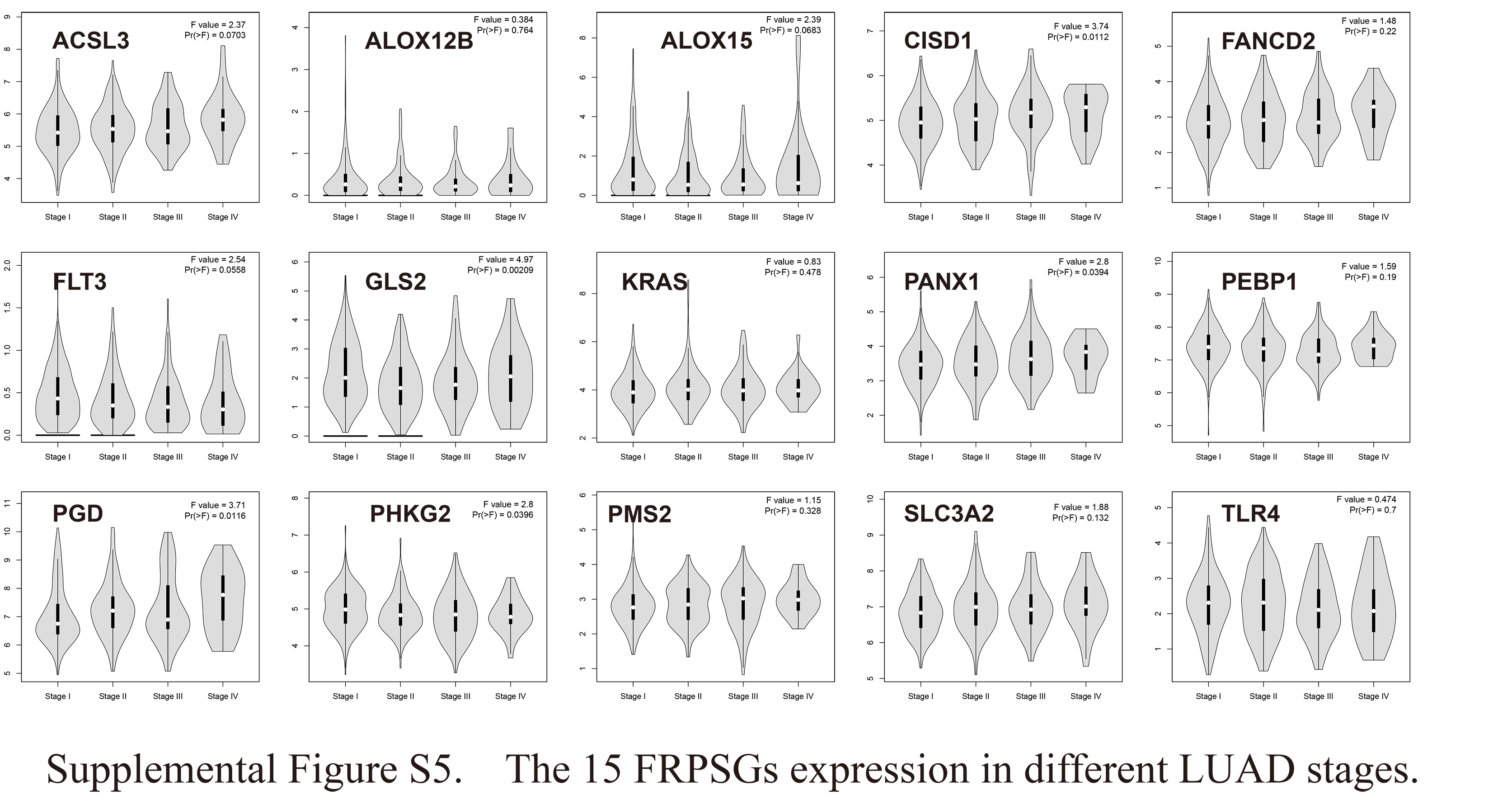

Supplement: Supplementary file 6 [file Image_6.TIF]

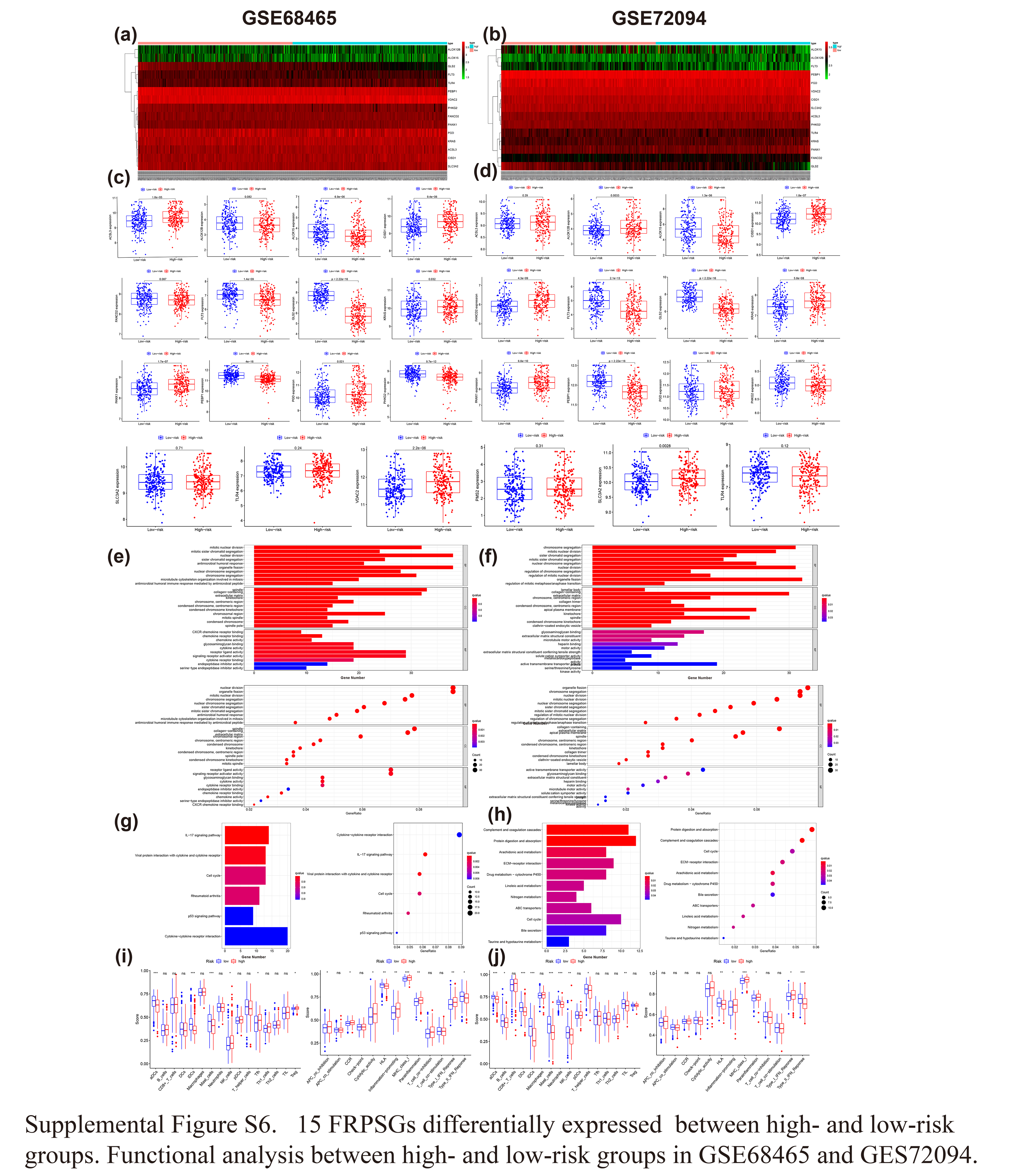

Supplement: Supplementary file 7 [file Image_7.TIF]

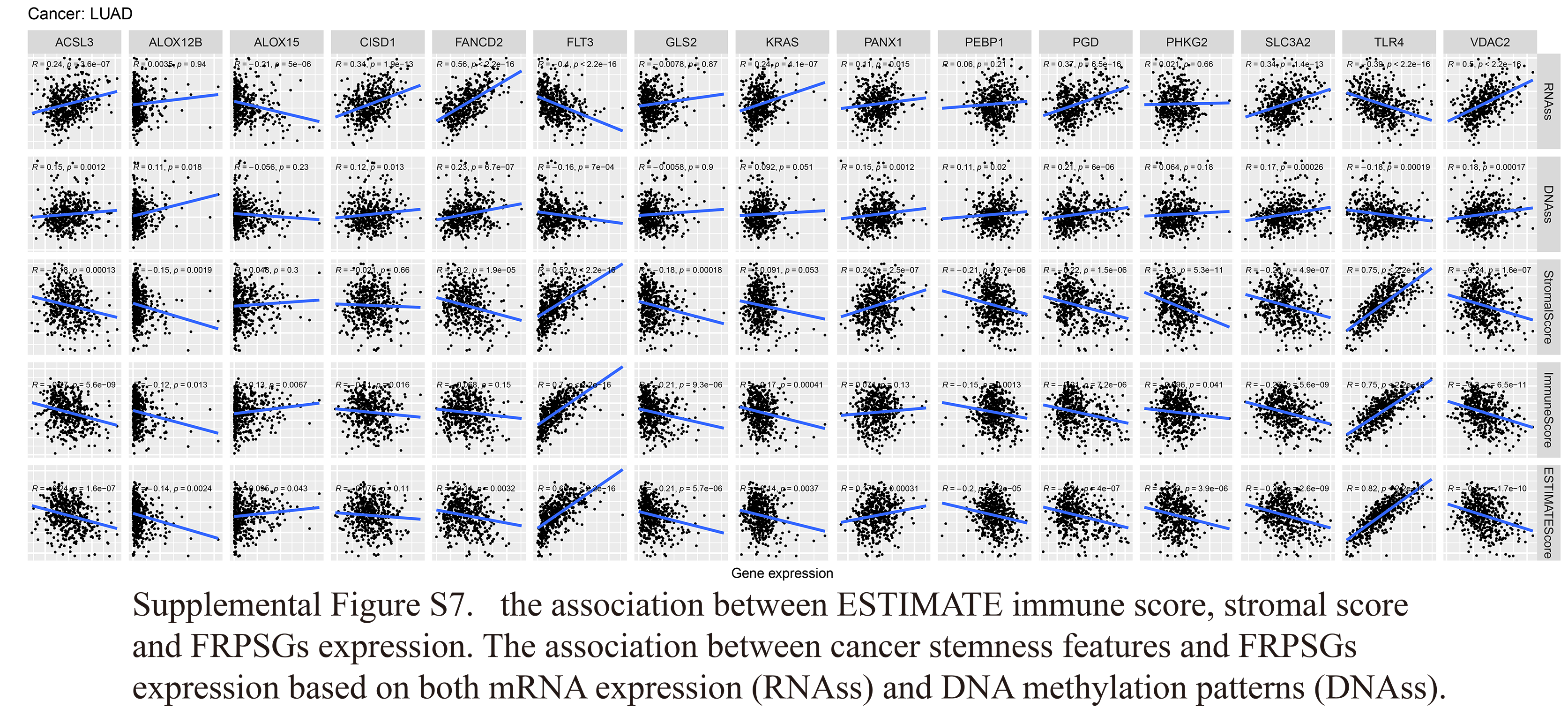

Supplement: Supplementary file 8 [file Image_8.TIF]

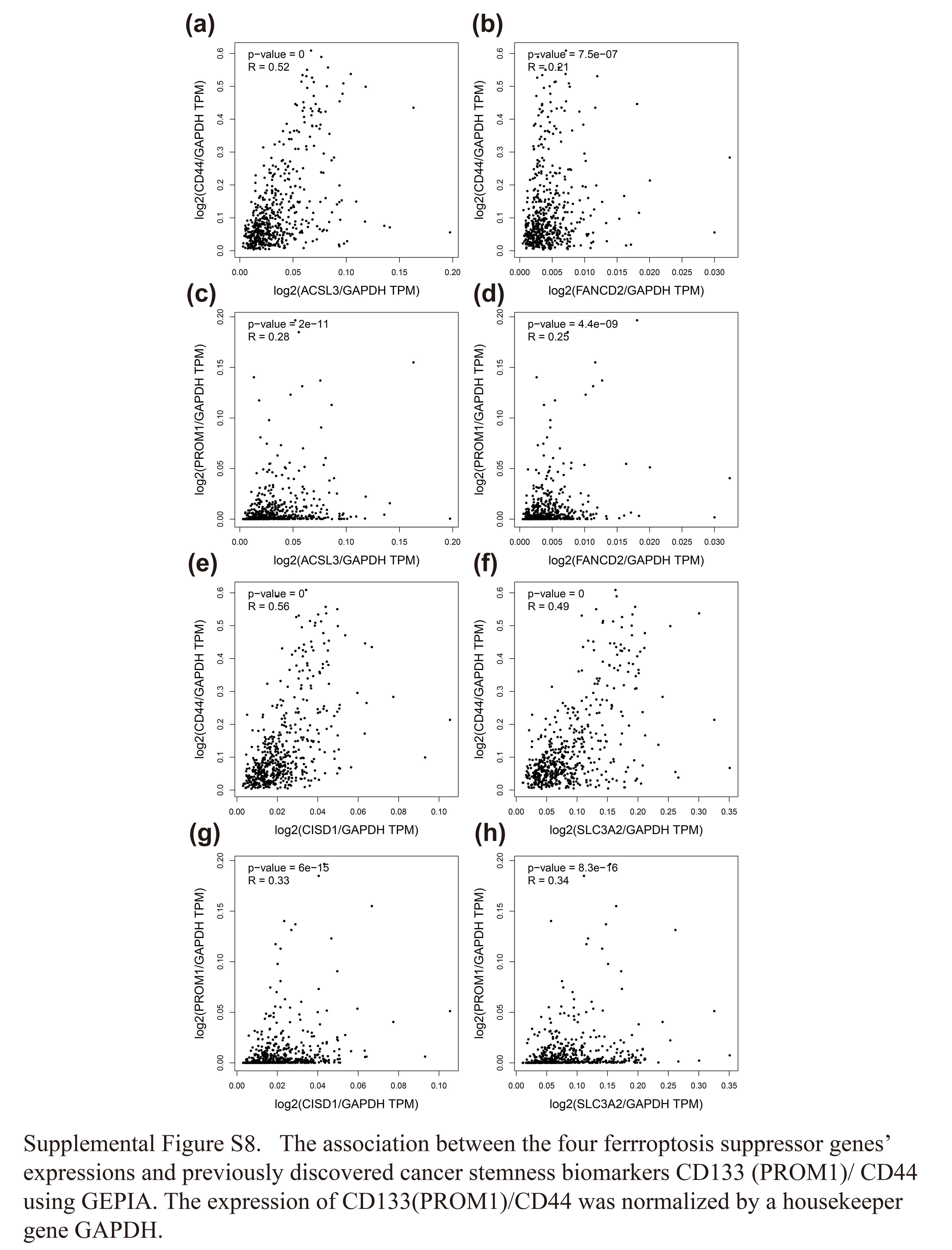

Supplement: Supplementary file 9 [file Image_9.TIF]
